# NUMBL Interacts with TAK1, TRAF6 and NEMO to Negatively Regulate NF-κB Signaling During Osteoclastogenesis

**DOI:** 10.1038/s41598-017-12707-7

**Published:** 2017-10-03

**Authors:** Gaurav Swarnkar, Tim Hung-Po Chen, Manoj Arra, Amjad M. Nasir, Gabriel Mbalaviele, Yousef Abu-Amer

**Affiliations:** 1 Department of Orthopaedic Surgery and Cell Biology & Physiology, Washington University School of Medicine, St. Louis, MO 63110 USA; 2 Bone and Mineral Division, Department of Medicine, Washington University School of Medicine, St. Louis, MO 63110 USA

## Abstract

NF-κB signaling is essential for osteoclast differentiation and skeletal homeostasis. We have reported recently that NUMB-like (NUMBL) protein modulates osteoclastogenesis by down regulating NF-κB activation. Herein, we decipher the mechanism underlying this phenomenon. We found that whereas NUMBL mRNA expression decreases upon stimulation of wild type (WT) bone marrow macrophages (BMMs) with RANKL, TAK1 deficiency in these cells leads to increased NUMBL and decreased TRAF6 and NEMO expression. These changes were restored upon WT-TAK1 expression, but not with catalytically inactive TAK1-K63W, suggesting that TAK1 enzymatic activity is required for these events. Forced expression of NUMBL inhibits osteoclast differentiation and function as evident by reduction in all hallmarks of osteoclastogenesis. Conversely, NUMBL-null BMMs, show increased osteoclast differentiation and mRNA expression of osteoclast marker genes. Post-translationally, K48-linked poly-ubiquitination of NUMBL is diminished in TAK1-null BMMs compared to elevated K48-poly-ubiquitination in WT cells, indicating increased stability of NUMBL in TAK1-null conditions. Further, our studies show that NUMBL directly interacts with TRAF6 and NEMO, and induces their K48-poly-ubiquitination mediated proteasomal degradation. Collectively, our data suggest that NUMBL and TAK1 are reciprocally regulated and that NUMBL acts as an endogenous regulator of NF-κB signaling and osteoclastogenesis by targeting the TAK1-TRAF6-NEMO axis.

## Introduction

Bone is a dynamic tissue which is constantly remodeled by the balanced activity of bone forming osteoblast (OB) and bone resorbing osteoclast (OC) cells. Many pathological and inflammatory conditions alter this homeostatic balance in favor of heightened OC differentiation leading to increased bone resorption and bone loss^1^. Various cellular signaling pathways have been studied in relation to OC differentiation and function. NF-κB, which was initially studied as a modulator of innate and adaptive immunity, has been shown as one of the most widely and important signaling pathway that regulates OC differentiation^2–4^. Upon binding of RANKL to its cognate receptor RANK, various adaptor proteins, including Tumor Necrosis Factor Receptor Associated Factor 6 (TRAF6), TGF-β Activated Kinase-1 (TAK1) and TAK1-Associated Adapter Protein 2 (TAB2), are recruited to RANK through ubiquitination and phosphorylation events, to form a signaling complex. This signaling complex further activates the IKK2 complex, which is comprised of IKK1, IKK2 and IKKγ/NEMO. The kinase activity of this complex phosphorylates IκB leading to its proteasome-mediated degradation and nuclear translocation of free NF-κB subunits, p65/RelA and p50 to the nucleus, resulting in activation and transcription of various genes essential for osteoclastogenesis^2,5–14^.

Multiple pathways including, PI3K^15^, Src^16^, MAPK^15^, PLCγ2^17,18^, NOTCH^19^ and Calcium/calcenurin^20^ contribute to osteoclastogenesis, yet NF-κB activation is a critical signaling pathway, absence of which impairs OCs differentiation entirely^21^. Accordingly, NF-κB signaling is considered crucial for maintaining skeletal homeostasis, perturbation of which leads to numerous pathologies. Hence, a greater interest has emerged to uncover and understand how NF-κB signaling interacts with other cellular circuits and the mechanisms via which they modulate NF-κB activity in health and disease. To this end, various regulators that interact with and regulate NF-κB signaling during basal and pathologic osteoclastogenesis have been identified.

TAK1 activates NF-κB through phosphorylation of IKK2 following stimulation with various stimuli, including RANKL, IL-1β and TNFα. The process involves recruitment of TAK1 to the NF-κB signalosome through adaptor proteins, such as TAB2 and polyubiquitin chains that facilitate arrangement of signaling proteins including TRAF6, NEMO, at close proximity. Using gene deletion studies, we have shown recently that TAK1 and NEMO are both essential for NF-κB activation and osteoclastogenesis, as absence of either protein enabled osteopetrosis^5,7^. Mechanistically, we have shown that TAK1 regulates expression of NUMB-like (NUMBL) and subsequently interact with Notch intracellular domain (NICD)/RBPJ signaling pathway during OC differentiation^7^.

NUMB and NUMBL have been studied as evolutionary conserved proteins which play a role in cellular fate determination during development^22,23^. However, the role of these proteins as TAK1 targets and regulators of NF-κB is poorly understood and their role in osteoclastogenesis is unknown. To this end, we have shown that deletion of TAK1 is associated with simultaneous increased expression of NUMBL and decreased expression of TAK1 adaptor protein TAB2. The increased NUMBL expression induces degradation of NICD, resulting in accumulation of co-repressor RBPJ that blunts NFATc1 expression and OC differentiation^7^. However, the interaction of NUMBL with TAK1/TAB2 and other components of NF-κB signaling during OC differentiation have not been fully elucidated.

In this regard, transfection studies have pointed to possible regulation of NF-κB signaling through interaction of NUMBL with TAB2 and TRAF6 in neurons^24,25^. Another report suggests that NUMBL regulates glioma cells migration and invasion by inhibiting TRAF5-induced NF-κB activation^26^. Based on these reports and our finding that NUMBL is involved in OC differentiation^7^, we herein studied the mechanism of NUMBL mediated regulation of NF-κB signaling and OC differentiation.

We found that during osteoclastogenesis, NUMBL expression is regulated at the transcriptional and translational levels. Our data indicate that RANKL stimulation decreases NUMBL mRNA expression in TAK1 sufficient bone marrow cells. It further suggests that TAK1/TAB2 complex mediates poly-ubiquitination of NUMBL marking it for proteasomal degradation. We also found that increased NUMBL expression leads to a concomitant decrease in TRAF6 and NEMO expression which results in inhibition of NF-κB activation and reduced OC differentiation and function. In summary, our data establishes NUMBL as a negative regulator of NF-κB signaling acting via interaction with TRAF6-TAB2-TAK1 complex and leading to regulation of the NICD-RBPJ axis, as we have shown recently^7^. Additional studies investigating the transcriptional control of NUMBL during OC differentiation will provide further understanding the mechanism of role of NUMBL in regulating bone homeostasis.

## Results

### TAK1 deletion leads to increased expression of NUMBL and decreased expression of TRAF6 and NEMO

**Figure 1:**
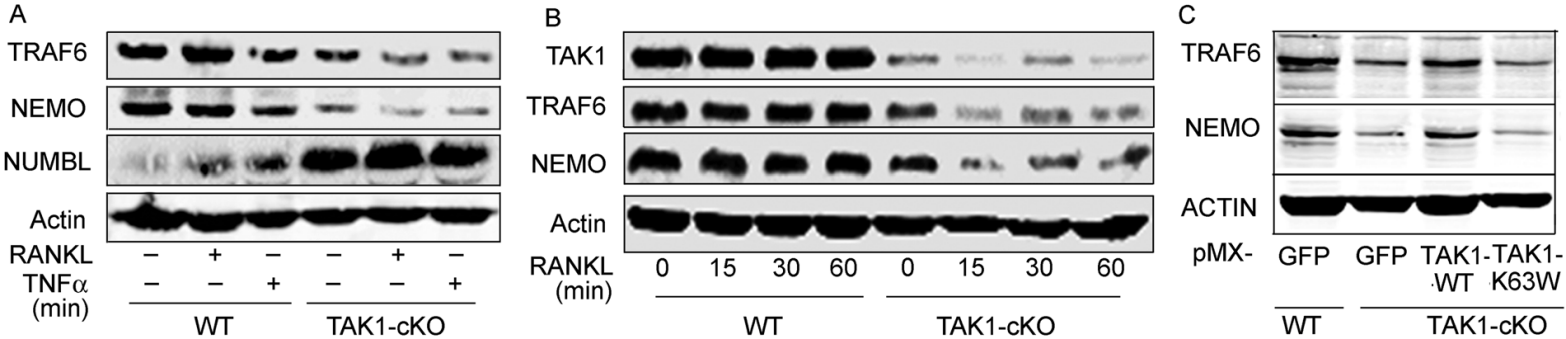
TAK1 deletion leads to increased expression of NUMBL and decreased expression of TRAF6 and NEMO, which gets rescued by exogenous expression of WT-TAK1. Bone marrow cells were isolated from WT or TAK1-cKO mice and cultured in presence of M-CSF. (**A**) Bone marrow cells were treated with or without RANKL and TNFα for 3 days in the presence of M-CSF. Western blots shows Increased NUMBL expression along with decreased TRAF6 and NEMO expression in TAK1-cKO cells when compared with WT cells. (**B**) WB showing decreased TRAF6, NEMO and TAK1 expression in TAK1-cKO cells compared to WT bone marrow cells, after short-term RANKL treatments. (**C**) Bone marrow cells were isolated from WT or TAK1-cKO mice were transfected with pMX-GFP, pMX-TAK1 and pMX-K63W and cultured in presence of M-CSF for 3 days followed by western blotting. WB showing rescue of TRAF6 and NEMO expression in TAK1-cKO cells expressing TAK1-WT but not in TAK1-cKO cells expressing catalytically inactive TAK1-K63W. Full-length Western blots images are included in supplementary data as Figure S1.A-C.

We have shown previously that conditional deletion of TAK1 in the myeloid lineage plummets RANKL-induced OC differentiation and that this process is associated with elevated expression of NUMBL concurrent with reduced NF-κB activation^7^. We also showed that combined deletion of NUMB/NUMBL form TAK1-cKO mice partially restores the osteopetrotic phenotype of TAK1-null mice and osteoclastogenesis^7^. Here we examined the possibility that NUMBL plays a repressor role during osteoclastogenesis. Specifically, we examined a potential mechanistic correlation between NUMBL expression and the key OC differentiation pathway encompassing TRAF6 and downstream NF-κB molecules. To this end, wild type (WT) and TAK1-null bone marrow cells were isolated and treated with vehicle, RANKL and/or TNF-α as indicated in Fig. 1A and B. Expression of TRAF6 and NEMO is stable in WT cells, but is significantly reduced in TAK1-null cells when equal total protein is loaded from the various conditions (confirmed by β-actin expression) (Fig. 1A and B). In contrast, expression of NUMBL is highly elevated in TAK1-null cells (Fig. 1A). This observation suggests that the divergent expression of NUMBL compared with TRAF6 and NEMO intersects at TAK1, and TAK1 not only mediates expression of TRAF6 and NEMO, but also regulates expression of potential repressors such as NUMBL.

### Expression of pMX-TAK1 inhibits NUMBL and restores TRAF6 and NEMO expression

To experimentally confirm that TAK1 indeed negatively regulates NUMBL, we examined the effect of forced expression of retroviral TAK1 in bone marrow cells. Our previous data show that WT (pMX-TAK1-WT) but not catalytically inactive TAK1 (pMX-TAK1-K63W) inhibits NUMBL expression concurrent with increased expression of TAB2 in in TAK1 null cells^7^. Under similar experimental conditions we observed that TAK1-WT but not TAK1-K63W rescues TRAF6 and NEMO expression in TAK1 null cells (Fig. 1C). These observations suggest that NUMBL directly or indirectly suppresses TRAF6 and NEMO expression and that TAK1 inhibits this action. More importantly, the data point out that NUMBL-induced repression of TRAF6 and NEMO in TAK1-null cells is reversible upon reconstitution of active TAK1 expression.

### NUBML acts as a negative regulator of osteoclastogenesis

**Figure 2:**
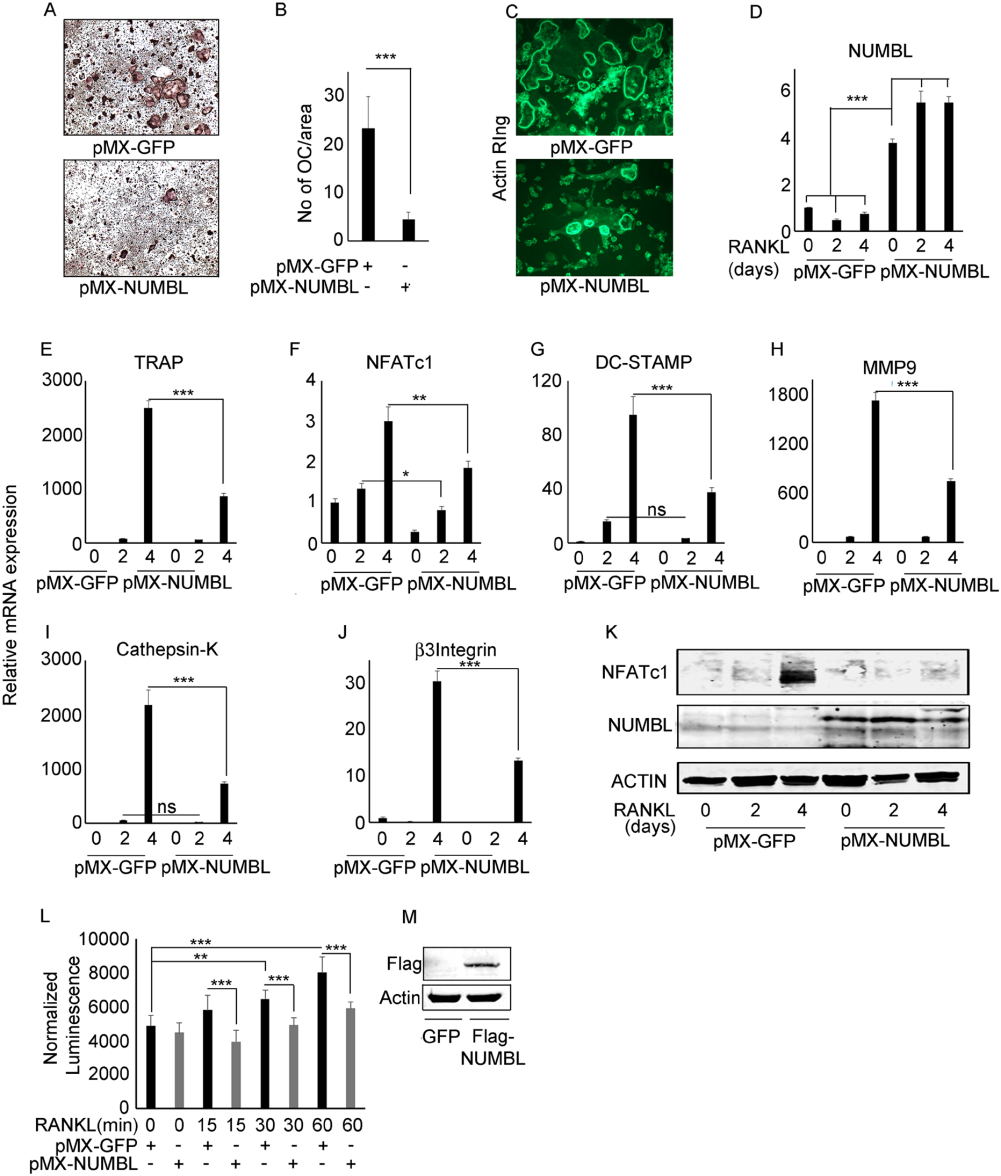
NUBML act as a negative regulator of osteoclastogenesis via inhibiting NF-κB activation. Bone marrow cells were isolated from WT mice and transfected with pMX-GFP and pMX-NUMBL. Two day after transfection, BMMs cells were plated in different plates and treated with RANKL and M-CSF for osteoclastogenesis, actin ring staining, mRNA isolation and western blot. (**A**,**B**) TRAP staining and (**C**) actin ring formation assay shows that NUMBL expression inhibits OC differentiation. (**D**) Increased NUMBL expression results in decreased expression of OC differentiation associated genes (**F**) TRAP, (**F**) NFATc1, (**G**) DC-STAMP, and (**H**) MMP9, (**I**) Cathepsin-K and (**J**) β3-Integrin in bone marrow cells upon RANK stimulation when compared with GFP expressing cells. (**K**) Western blots suggest decreased NFATc1 expression in NUMBL overexpressing cells in response to RANKL stimulation when compared with GFP expressing control cells. β-Actin was used as control for both qPCR and Western blotting. Full-length Western blots images are included in supplementary data as Figure S2.K. (**L**) Bone marrow cells isolated from RelA-Luc reporter mice were transfected with pMX-GFP and pMX-NUMBL and cultured in presence of M-CSF for 3 days, followed by RANKL stimulation for 0, 15, 30 and 60 minutes. RelA-luciferase activity measurement shows that NUMBL overexpression results in decrease in NF-κB activation in comparison to GFP expressing cells upon RANKL stimulation. (**M**) NUMBL expression was confirmed by western blotting for Flag. (**P < 0.01 and ***P < 0.001).

Our data suggest that TAK1 deletion leads to increased NUMBL expression with concomitant decrease in TRAF6 and NEMO expression. Based on this finding, we hypothesized that NUMBL acts as a negative regulator of osteoclastogenesis. To test this proposition, WT bone marrow cells were transfected with pMX-GFP and pMX-NUMBL and subjected to osteoclastogenesis, actin ring formation, as well as qPCR and biochemical analyses. Our data show that NUMBL overexpression decreases RANKL induced OC differentiation (Fig. 2A,B) and fewer actin rings on bone slices (Fig. 2C). Further, qPCR data show that NUMBL over-expression results in decreased mRNA expression of TRAP, NFATc1, DC-STAMP, MMP9, β3-integrin and cathepsin-K. (Fig. 2D,J). Western blot analysis also shows a decrease in NFATc1 expression in response to RANKL treatment of pMX-NUMBL overexpressing cells (Fig. 2K). To confirm the effect of increased NUMBL expression on NF-κB activation, RelA-Luc reporter assay was performed using bone marrow cells isolated from RelA_luc-reporter mice. The luminescence activity shows that NUMBL expression decreases NF-κB activation in response to RANKL treatment when compared with control GFP expressing cells (Fig. 2L). Expression of transduced pMX-flag-NUMBL was confirmed by western blot using FLAG antibody (Fig. 2M). This data suggest that increased NUMBL expression inhibits OC differentiation.

### Deletion of NUMBL in myeloid lineage leads to increase in osteoclastogenesis

**Figure 3:**
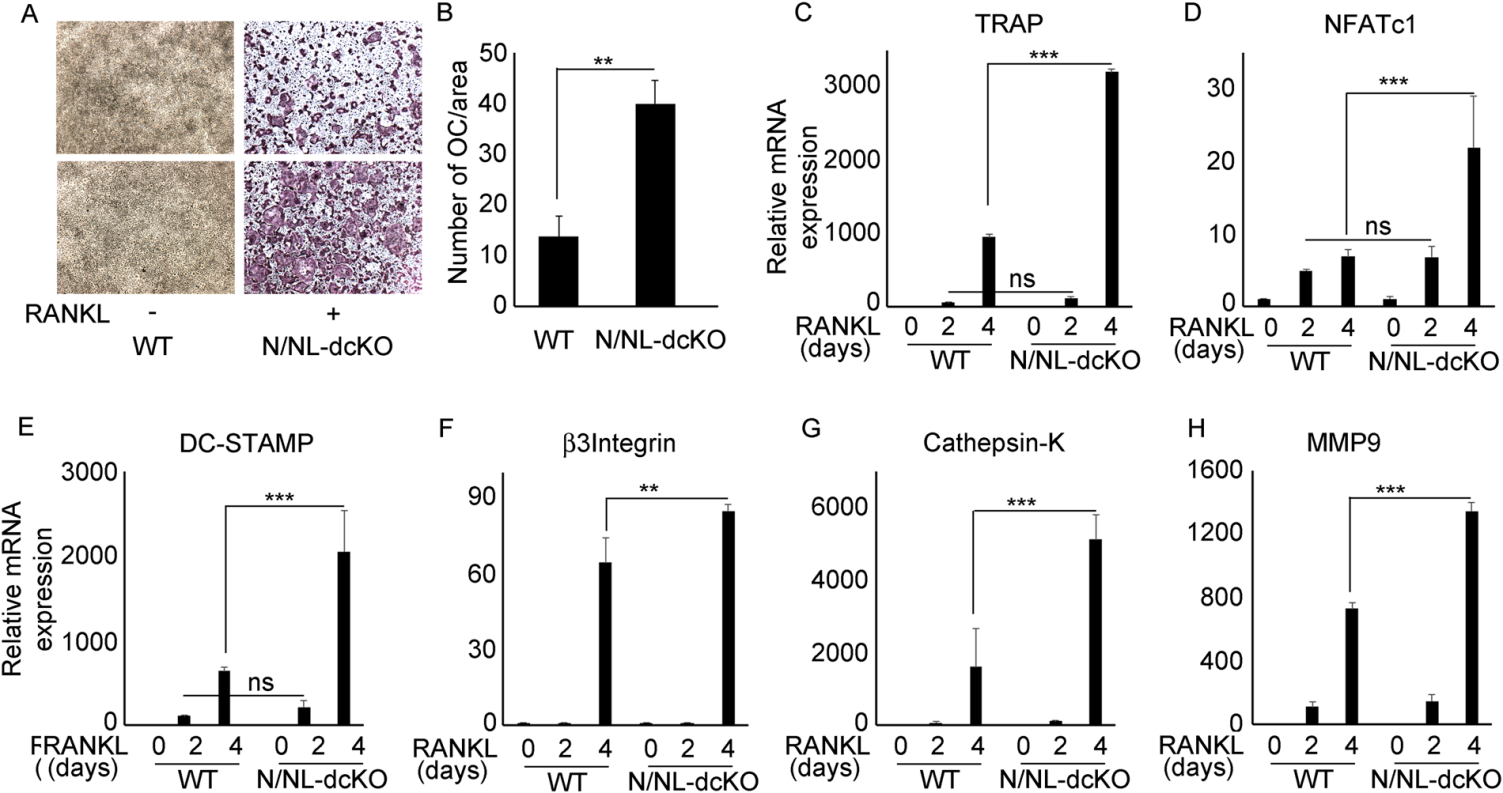
Deletion of NUMBL in myeloid lineage leads to increase in osteoclastogenesis. Bone marrow cells were isolated from WT and NUMBL-cKO mice. (**A**) Cells were cultured in 96 well plate in the presence of RANKL and M-CSF for 4 day followed by fixation, TRAP staining and (**B**) counting. Bone marrow cells from WT and NUMBL-cKO mice were treated with RANKL for 0, 2 and 4 days in the presence of M-CSF, followed by RNA isolation for qPCR of OC differentiation associated genes. NUMBL deletion results in increased expression of OC differentiation associated genes (**C**) TRAP, (**D**) NFATc1, (**E**) DC-STAMP, (**F**) β3-Integrin, (**G**) Cathepsin-K and (**H**) MMP9 in bone marrow cells upon RANK stimulation. (**P < 0.01 and ***P < 0.001).

To further investigate the effect of NUMBL on basal OC differentiation, bone marrow cells from WT and Numb/Numbl double knockouts (N/NL-dcKO) mice (as detailed in the methods section) were differentiated to osteoclasts in the presence of RANKL and M-CSF for 4 days. We decided to use cells from mice lacking both NUMB and NUMBL to avoid any potential redundancy of NUMB. The results indicate that deletion of NUMBL leads to increased OC differentiation (Fig. 3A,B). This observation is further supported by increased mRNA expression of OC differentiation associated genes TRAP, NFATc1, DC-STAMP, β3-integrin, cathepsin-K and MMP9 (Fig. 3C–H).

### RANKL inhibits NUMBL expression and induces NUMBL K48-linked poly UB in TAK1-dependent manner

**Figure 4:**
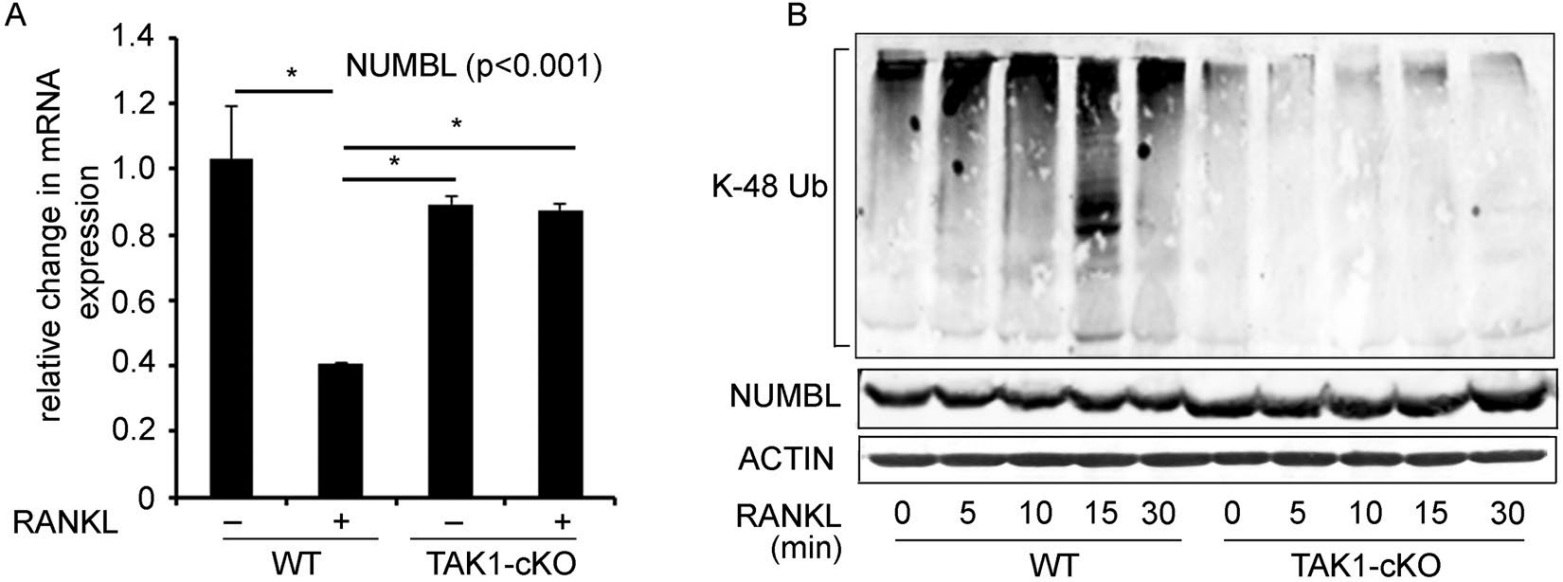
RANKL inhibits NUMBL expression and induces NUMBL K48-poly UB in TAK1-dependent manner. (**A**) Bone marrow cells, from WT and TAK1-cKO mice were cultured in presence or absence of RANKL and M-CSF for 1 day followed by mRNA isolation and qPCR for NUMBL. RANKL treatment significantly decreases NUMBL expression but in WT cells but failed to inhibit NUMBL expression under TAK1 deletion condition. (**B**) Bone marrow cells from WT and TAK1-cKO mice were treated with RANKL as indicated. Total cell lysates and NUMBL-immunoprecipitates were then immunoblotted with anti-NUMBL and anti-K48-UB specific antibodies, respectively. Hypo K48-polu-Ubiquitinaion in TAK1-cKO cells indicates that TAK1 mediates poly-ubiquitination of NUMBL destined for proteasome degradation. (*P < 0.05).

Based on our data, we next studied potential mechanisms underlying TAK1-dependent regulation of NUMBL during RANKL-induced OC differentiation. To understand the mechanism of NUMBL inhibition, we measured the NUMBL mRNA and protein expression in RANKL stimulated bone marrow cells. Contrary to increases observed in NUMBL protein expression after 3–4 days of RANKL treatment (Fig. 1A), WT bone marrow cells treated with RANKL for 1 day in the presence of M-CSF show decrease in NUMBL mRNA expression (Fig. 4A). In contrast, in TAK1-null cells, RANKL stimulation failed to inhibit NUMBL mRNA expression (Fig. 4A). Next, to investigate if TAK1 regulates NUMBL expression post-translationally, we measured ubiquitination of NUMBL in immunoprecipitates from vehicle or RANKL-treated WT and TAK1cKO-derived bone marrow cell lysates. The data suggest that TAK1 deletion leads to hypo-K48-linked poly UB of NUMBL, which stabilizes its expression. These observations along with preivious data showing decreasesed NUMBL and increased TAB2 expression in TAK1-null expressing TAK1-WT but not catalytically inactive TAK1-K63W^7^, suggest that under normal conditions, TAK1 activity and presence of TAK1-TAB2 signaling may mediate poly-ubiquitination of NUMBL leading to its proteasomal degradation (Fig. 4B).

### NUMBL overexpression decreases TRAF6 and NEMO abundance by inducing their K48-linked poly-Ubiquitination

**Figure 5:**
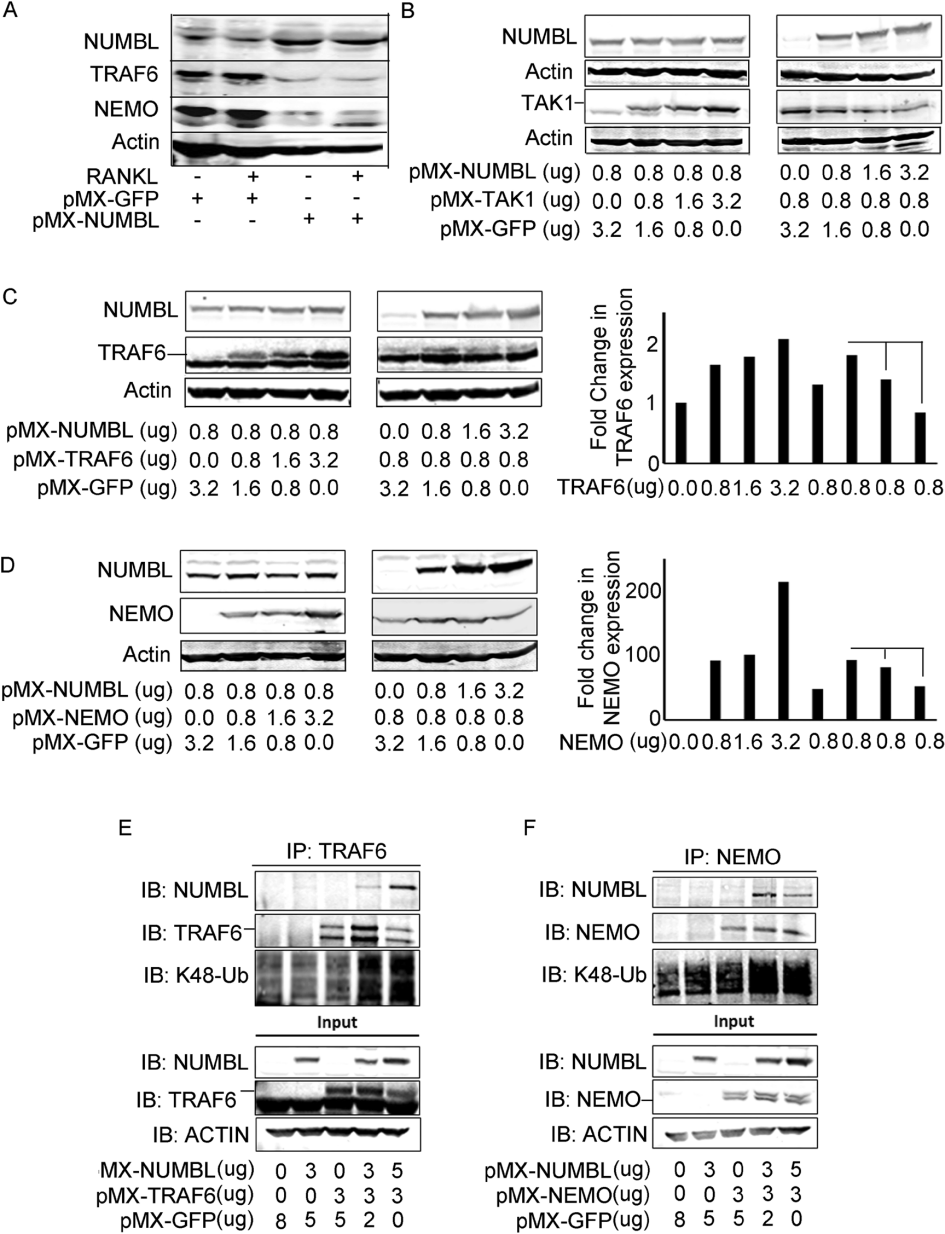
NUMBL overexpression inhibits TRAF6 and NEMO by inducing K48-pUB of TRAF6 and NEMO. (**A**) WT bone marrow cells expressing pMX-NUMBL showed decreased expression of TRAF6 and NEMO upon RANKL stimulation when compared with pMX-GFP expressing cells after 2 days of RANKL stimulation. Plat-E cells were co-transfected with fixed amount of pMX-NUMBL in the presence of increasing amount of (B; left panel) TAK1, (C; left panel) TRAF6 and (D; left panel) NEMO or with increasing amount of pMX-NUMBL with fixed amount of (B; right panel) TAK1, (C; middle panel) TRAF6 and (D; middle panel) NEMO. 2 days after transfection the cells were lysed and immuno-probed with indicated antibodies. Immuno-blots indicate that increasing NUMBL expression results in decrease in TAK1, TRAF6 and NEMO expression in Plat-E cells. The expression of (C: right panel) TRAF6 and (D; right panel) NEMO was quantitated using image J. (**E**) Plat-E cells were co-transfected with NUMBL and TRAF6. Immuno-blot from total cell lysates and TRAF6-immunoprecipitates shows that NUMBL interacts with TRAF6 and leads to its K48-poly-ubiquitine mediated proteasomal degradation. (**F**) Plat-E cells were co-transfected with NUMBL and NEMO. Immuno-blot from total cell lysates and NEMO-immunoprecipitates shows that NUMBL interacts with TRAF6 and leads to its K48-poly-ubiquitine mediated proteasomal degradation. Full-length Western blots images are included in supplementary data as Figure S5.A-F.

Our results indicate that RANKL-induced NF-κB activation restrains NUMBL expression and that NUMBL negatively regulates NF-κB activation most likely by targeting TRAF6 and NEMO, both essential for NF-κB activation. To further interrogate this potential mechanism, we treated GFP- and NUMBL-expressing WT bone marrow macrophages with RANKL for 4 days followed by Western blot for TRAF6 and NEMO. We find that NUMBL overexpression results in significant decrease in TRAF6 and NEMO expression (Fig. 5A). As endogenous expression of TRAF6, NEMO and TAK1 is not sufficient to carry out co-immmunoprecipitation and ubiquitination studies since increased NUMBL expression (in TAK1-null cells) will degrade the TRAF6 and NEMO, we employed transfection studies to confirm the NUMBL-mediated degradation of TRAF6, NEMO and TAK1 in Plat-E cells (modified HEK293 cells). To this end, Plat-E cells were co-transfected with increasing amount of TAK1, TRAF6, and NEMO with constant amount of NUMBL or with constant amount of TAK1, TRAF6, and NEMO in the presence of increasing amount of NUMBL. Two day post transfection the cells were lysed and subjected to Western blotting. The results indicate that increasing concentrations of either TAK1, TRAF6 and NEMO do not affect NUMBL expression, but increasing NUMBL concentration results in decrease of TAK1 (Fig. 5B), TRAF6 (Fig. 5C), and NEMO (Fig. 5D) expression. This led us to speculate that NUMBL directly interacts with TRAF6 and NEMO and promotes their K48-pUB mediated proteasomal degradation. To verify this possibility, NUMBL-TRAF6 and NUMBL-NEMO co-transfected Plat-E cells were lysed and immunoprecipitated for TRAF6 and NEMO followed by immunoblotting for K48-pUB and NUMBL. For control, Plat-E cells were individually transfected with NUMBL, TRAF6 and NEMO and immunoprecipitated for TRAF6 and NEMO. Co-immunoprecipitation first shows that NUMBL interacts with TRAF6 (Fig. 5E) and NEMO (Fig. 5F). Abundance of K48-pUB in TRAF6, NEMO and NUMBL transfected cells when compared with cells co-transfected with TRAF6, NEMO combination only shows that NUMBL induces K48-pUB mediated proteasomal degradation of TRAF6 (Fig. 5E) and NEMO (Fig. 5F). The observation that TAK1 overexpression does not affect NUMBL levels (Fig. 5B, left panel), suggests that additional mechanisms regulate baseline NUMBL expression. In this regard, TAK1-mediated degradation of NUMBL may require other interacting partners, which were not over-expressed in Plat-E cells but otherwise have normal expression in primary cells.

## Discussion

NF-κB signaling is crucial to maintain cellular homeostasis in all cell types including OCs and their myeloid precursors. During differentiation of myeloid progenitors into OCs, RANKL triggers formation of a signaling complex encompassing TAK1, TRAF6 and other adaptor proteins that attach to distal motifs of RANK culminating with stimulation of NF-κB signaling. This process eventually leads to induction of transcriptional machinery for OC formation that contains several activators and repressors.

Various regulators of NF-κB signaling have been identified in OCs and other cells, which interact with key players of NF-κB signaling to maintain cellular homeostasis. For example, A20^27^, IRF8^28^, Bcl6^29^, RBPJ^5,7,30,31^ have been reported to repress NF-κB signaling during OC differentiation. Other regulators such as PARPs, were shown to upregulate NF-κB activation in OC and other cells types^32–34^. Hence, better understanding of molecules and systems that regulate RANKL-induced NF-κB signaling and osteoclastogenesis will offer new insights to regulate this pathway and maintain skeletal health.

In a previous study, we discovered that NUMBL, which is important for determination of cell fates during development, is involved in regulating the osteoclastogenic machinery. Specifically, under TAK1 deficient conditions, elevated levels of NUMBL inhibit OC differentiation by degrading Notch intracellular domain (NICD). This event leads to accumulation of the transcription factor RBPJ, resulting in transcriptional repression of NFATc1^7^. In this current study, we show that NUMBL directly represses NF-κB activation during RANKL-induced OC differentiation by regulating proximal components of NF-κB signaling.

We first observed that increased NUMBL expression in TAK1 null cells coincides with decreased TRAF6 and NEMO expression, both of which are key components of RANKL mediated NF-κB signaling. Moreover, exogenous expression of catalytically active TAK1 not only decreases NUMBL^7^ expression but also restores TRAF6 and NEMO expression in TAK1 null cells, suggesting that TAK1, TRAF6 and NEMO may interact with NUMBL and reciprocally regulate OC differentiation, where NUMBL might be acting as a negative regulator of NF-κB activation. NUMBL overexpression studies in BMMs clearly suggest that NUMBL inhibits OC differentiation and function via inhibiting NF-κB activity and NFATc1 expression. Conversely, NUMBL deficient cells exhibit increased OC differentiation and expression of genes associated with OC differentiation. These experiments establish NUMBL as a negative regulator of OC differentiation and function.

NUMBL has been shown to interact with TAB2 (TAK1 binding protein 2) and mediates TRAF6 degradation, thus inhibiting NF-κB activation^24,25^. In glioma cells NUMBL has been reported as a suppressor of TRAF5 mediated NF-κB activation^25^. Based on this and our data, we hypothesized that RANKL-mediated NUMBL regulation during OC differentiation is TAK1-dependent. Gene expression data suggest that in TAK1 sufficient WT cells, RANKL inhibits NUMBL transcription during early stages of RANKL stimulation. At the same time detection of K48-linked ubiquitination in immuno-precipitates using NUMBL antibody in WT and TAK1-null cells shows that, whereas in WT cells TAK1 leads to K48-linked pUB mediated degradation of NUMBL, in TAK1-null cells NUMBL accumulates due to hypo-K48-ubituitiantion and, as a result, reduced degradation. As we have suggested previosuly, NUMBL expression is TAK1-dependent and exerts a baseline osteoclast regulation when expressed. Hence it is possible that RANKL exerts a temporal effect, wherein at early stages of RANKL stimulation TAK1 is activated leading to reduced NUMBL expression and initiation of osteoclastogenesis. As this osteoclastogenesis process is established, TAK1 activity is reduced by feedback mechanisms which coincide with increased expression of osteoclast negative feedback mechanisms, i.e. increased NUMBL expression. Further studies are required to establish the mechanism by which RANKL treatment decreases NUMBL expression at the transcriptional level and TAK1 kinase activity mediated-NUMBL degradation post translationally. It is likely that RANKL-induced TAK1 forms a complex with TAB2 and TRAF6, a well-known E3 ligase, or other yet to be identified ligases to induce ubiquitination of NUMBL. We further speculate that TAK1 may target the inducible expression of NUMBL which is presumably post-transnationally modified and marked for ubiquitination mediated degradation as opposed to the basal levels of un-modified NUMBL.The molecular details of such mechanism await further studies.

To further understand the mechanism of NUMBL-mediated NF-κB regulation, we analyzed TRAF6 and NEMO expression in WT BMMs cells over-expressing NUMBL. Immunoblots suggest that increased NUMBL expression in WT cells is associated with decreased TRAF6 and NEMO expression. As described earlier, NUMBL acts as an E3 ligase and mediates degradation of proteins like TRAF6^24^. Based on this, our overexpression study in Plat-E cells shows that increasing expression of NUMBL leads to decreased expression of TAK1, TRAF6 and NEMO. However, increasing expression of TAK1, TRAF6 or NEMO do not drive down the expression of NUMBL below its normal baseline level. One possible explanation is that TAK1 interaction with NUMBL requires other proteins such as TAB2 and ligases. Given the fact that TAB2 is also an obligatory TAK1 partner, it is possible that TAB2 is a rate-limiting protein that determines the overall homeostatic balance between TAK1 and NUMBL function under RANKL-induced conditions, e.g. NF-κB activation and OC differentiation.

The evidence that NUMBL physically interacts with both TRAF6 and NEMO, and induces their K48-ubiquitination mediated proteasomal degradation further supports its fundamental role as potential RANKL-induced NF-κB signaling repressor. This process is pronounced in the absence of TAK1, suggesting that NUMBL is an innate mechanism that dampens NF-κB activation and osteoclastogenesis at times when activation of TAK1 by RANKL subsides. Using the same rational, it is also possible that NUMBL plays a role in regulating other signaling complex featuring TRAF6 or TAK1 that are required for normal osteoclastogenesis. For example, NUMBL is predicted to interact with atypical-PKCs (a-PKC), which have been shown to form a complex which includes p62/TRAF6/a-PKC upon RANKL stimulation in BMMs^35^.

Since NUMBL expression is normally decreased or low under normal OC differentiation, it will be interesting to determine the expression and regulation of NUMBL in other pathological models of osteolysis such as arthritis. One can speculate that expression level of NUMBL remain at baseline during inflammatory conditions owing to constant activity of TAK1 and elevated expression and ligase activity of TRAF6. Therefore future studies are required to further elucidate the possible role of NUMBL under normal and pathological OC differentiation and explore the utility of this innate mechanism in osteolytic conditions.

Collectively, our data present NUMBL as a negative regulator of osteoclastogenesis. Increased NUMBL expression, in one hand, regulates NFATc1 expression via accumulating RBPJ (NICD/RBPJ axis). In the other hand, it directly induces K48-mediated proteasomal degradation of TRAF6 and NEMO to dampen NF-κB activation and attenuate osteoclast differentiation.

## Methods

### Animals

TAK1 floxed mice on a C57BL/6 background were crossed with LysM-Cre (Jacksons Laboratories) to produce heterozygous mice. The TAK1 heterozygous mice were further intercrossed to generate homozygous null mice (TAK1-cKO). NUMB floxed and NUMBL floxed mice were obtained from Jacksons Laboratories which were bred together to generate heterozygous mice followed by intercrossing to generate homozygous double floxed mice (NUMB/NUMBL floxed termed N/NL floxed). Later the double floxed mice were crossed with LysM-Cre to finally generate a NUMB/NUMBL double knockout (N/NL-dcKO mice). RelA_luc reporter mice were purchased from Taconic Biosciences. Mice were housed at the Washington University School of Medicine barrier facility. All experimental protocols were carried out in accordance with the ethical guidelines approved by the Washington University School of Medicine Institutional Animal Care and Use Committee.

### Cell Culture

Bone marrow cells were cultured in α-MEM supplemented with 100 units/ml penicillin/streptomycin and 10% FBS (v/v) with 10 ng/ml M-CSF for 16 h to separate adherent cells from non-adherent cells. Non-adherent cells were collected and further cultured with M-CSF (20 ng/ml) for 3 to 4 days, followed by RANKL (50 ng/ml) treatment for different time points (as described in the figures). In certain conditions, TAK1-cKO cells were either cultured with TNFα neutralizing antibody (0.2 μg/ml). To understand the interaction of NUMBL with TRAF6 and NEMO and to generate retroviral vectors Plat-E cells were cultured in DMEM supplemented with 100 units/ml penicillin/streptomycin and 10% FBS (v/v)^7^.

### Transfection and retroviral infection

For expression studies, various genes like NUMBL, TAK1-WT, TAK1-K63W, TRAF6 and NEMO were cloned in retroviral pMX- constructs. For exogenous expression, relevant pMX-constructs were transfected in Plat-E cells using xtrem gene9 (Roche,). Similarly, for retroviral production relevant pMX- constructs were first transfected into Plat-E cells using xtreme gene 9, followed by collection of virus containing media for 2 days. This virus containing media were later used to infect bone marrow cells^7^.

### Actin ring staining

Osteoclasts were generated on bone slices from bone marrow macrophages (BMMs) expressing GFP and NUMBL. For actin ring staining, the cells were fixed in 4% paraformaldehyde and permeabilized in 0.1% Triton X-100, rinsed in PBS, and immunostained with AlexaFluor 488-phalloidin (Invitrogen).

### RelA-Luc reporter assay

BMMs isolated form RelA_luc reporter mice (Taconic), were transduced with pMX-GFP and pMX-NUMBL retroviral particles. One day after transduction the cells were cultured in 96 well plate in the presence of M-CSF for two days, followed by starvation and RANKL treatment for 0, 15, 30 and 60 min. Post RANKL transfection cells were lysed and luciferase activity was measured using luciferase assay system (Promega). The luciferase activity was normalized with protein concentration (BCA assay, OD at 592 nm).

### Western blot Analysis

Total cell lysates were prepared in cell lysis buffer (Cell Signaling Technology, Danvers, MA, USA) from either Plat-E cells expressing different genes or Bone marrow cells induced with RANKL and/or TNFα for different time points as described in the figures). Protein concentration was determined and equal amount of protein was loaded onto SDS-PAGE. After transfer, and blocking in 5% BSA for 1 h at room temperature, membranes were probed with specific primary antibody primary antibodies in 5% BSA in PBS-Tween (1% v/v) for overnight and then washed three times with PBS-Tween (PBST) and probed with secondary antibodies from LI-COR (Odyssey Imager; donkey anti-rabbit/IRDye 800CW/anti-goat/IRDye 800CW anti-mouse IRDye 680RD) for 1 h at room temperature. Membranes were then washed three times with PBST and scanned by using LI-COR Odyssey Imager (LI-COR Biosciences, Lincoln, NE, USA). The NEMO, TRAF6, NUMBL, NFATc1 and TAK1 antibodies were purchased from Santa Cruz, Dallas, TX, USA; K48-Ub antibody was purchased from Cell Signaling Technology, Danvers, MA, USA; β-actin was purchased from Sigma, St Louis, MO, USA.

### qPCR analysis

Cells were cultured in presence of M-CSF (20 ng/ml) and RANKL (50 ng/ml) for 3 or 4 days as labeled in the figures. mRNA was isolated using PureLink RNA mini kit (Ambion, Grand Island, NY, USA) and cDNA were prepared using High Capacity cDNA Reverse Transcription kit (Applied Biosystems). qPCR was carried out on BioRad CFX96 real time system using iTaq universal SYBR green super-mix (BioRad, Hercules, CA, USA). mRNA expression were normalized using actin as a housekeeping gene. The following primers were used for qPCR analysis. TRAP-F: CGACCATTGTTAGCCACATACG, TRAP-R: CACATAGCCCACACCGTTCTC, CTSK-F: ATGTGGGTGTTCAAGTTTCTGC, CTSK-R: CCACAAGATTCTGGGGACTC, MMP9-F: ACTGGGCTTAGATCATTCCAGCGT, MMP9-R: ACACCCACATTTGACGTCCAGAGA, NFATc1-F: CCGGGACGCCCATGCAATCTGTTAGT, NFATc1-R: GCGGGTGCCCTGAGAAAGCTACTCTC, NUMBL-F: GCACGGACTTCCAGGTGA, NUMBL-R: CATTGATGCCATCGCTGT, DC-STAMP-F: ACAAACAGTTCCAAAGCTTGC, DC-STAMP-R: GACTCCTTGGGTTCCTTGCT, β3Integrin-F: GCAAGTACTGTGAGTGCGATG, β3Integrin-R: CGCAGTCCCCACAGTTACA.

### Co-Immuno precipitation

Plat-E cells (modified HEK293 cells) were co-transfected with pMX-NUMBL and pMX-TRAF6 or pMX-NEMO. Two days post-transfection the cells were washed with ice cold PBS and lysed in 1 ml of lysis buffer (10 mM Tris-HCL-pH-8.0, 1 mM EDTA, 125 mM NaCl and 1% NP-40) containing protease and phosphatase inhibitor (Halt Protease and phosphatase inhibitor, ThermoFischer Scientific). 200 ul of the total cell lysate was saved as total cell lysates for total loading control and protein estimation. Equal amount of protein from remaining lysates were incubated with 4ug of anti-TRAF6 or anti-NEMO antibody for 2 hours followed by addition of Protein-G beads (Genescript, NJ,US) for overnight at 4 °C. After overnight incubation the beads were washed three times with washing buffer (10 mM Tris-HCL-pH-8.0, 1 mM EDTA, 20 mM NaCl and 1% NP-40) containing protease and phosphatase inhibitor and resuspended in protein loading buffer followed by immuno-blot (IB) analysis of immuno-precipitated (IP) and total cell lysate (TCL) fractions.

### Statistical Analysis

All experiments were repeated at least three times. Statistical analyses were performed by using Student *t*-test. Multiple treatments were analyzed by using one-way ANOVA followed by post hoc Newman-Keuls test of significance. Values are expressed as mean ± SD of at least three independent experiments. *P* values are indicated where applicable.

## Electronic supplementary material

Supplementary info
