# Supplementary material for: NUMBL Interacts with TAK1, TRAF6 and NEMO to Negatively Regulate NF-κB Signaling During Osteoclastogenesis
Source: Sci Rep. 2017 Oct 3;7:12600. doi: 10.1038/s41598-017-12707-7 (PMC5626749; doi:10.1038/s41598-017-12707-7)
Supplement: Supplementary file 1 — Supplementary info [file 41598_2017_12707_MOESM1_ESM.pdf]

Supplementary Information

NUMBL Interacts with TAK1, TRAF6 and NEMO to Negatively Regulate NF-κB Signaling During Osteoclastogenesis

Gaurav Swarnkar[1], Tim Hung-Po Chen[1], Manoj Arra[1], Amjad M Nasir[1], Gabriel Mbalaviele[2], Yousef Abu-Amer*[1]

[1]Department of Orthopaedic Surgery and Cell Biology & Physiology, Washington University School of Medicine, St. Louis MO 63110
[2]Bone and Mineral Division, Department of Medicine, Washington University School of Medicine, St. Louis MO 63110

Figure S.1A

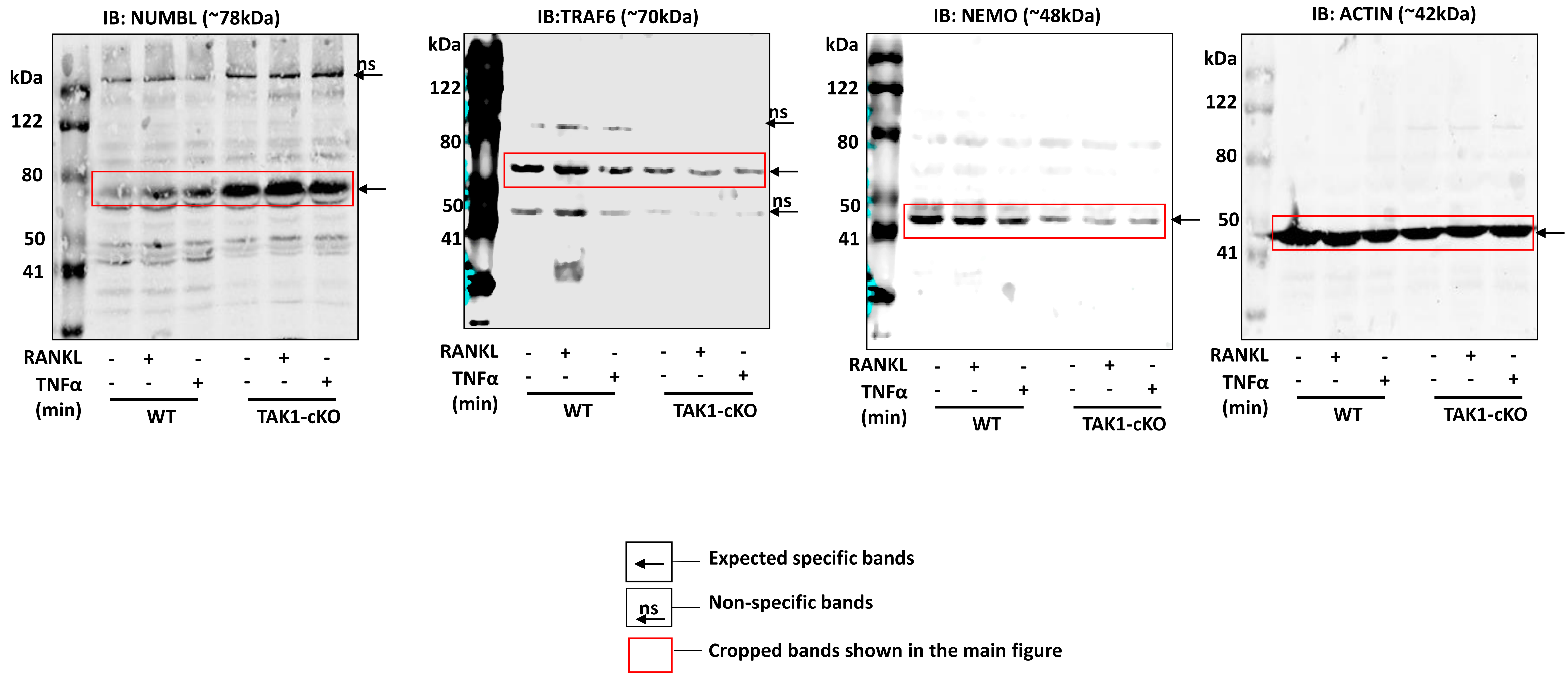

IB: NUMBL (~78kDa)
kDa
122
80
50
41
ns
RANKL - + - - + -
TNFα - - + - - +
(min)
WT
TAK1-cKO
IB:TRAF6 (~70kDa)
kDa
122
80
50
41
ns
ns
RANKL - + - - + -
TNFα - - + - - +
(min)
WT
TAK1-cKO
IB: NEMO (~48kDa)
kDa
122
80
50
41
RANKL - + - - + -
TNFα - - + - - +
(min)
WT
TAK1-cKO
IB: ACTIN (~42kDa)
kDa
122
80
50
41
RANKL - + - - + -
TNFα - - + - - +
(min)
WT
TAK1-cKO
Expected specific bands
ns
Non-specific bands
Cropped bands shown in the main figure

Figure S.1B

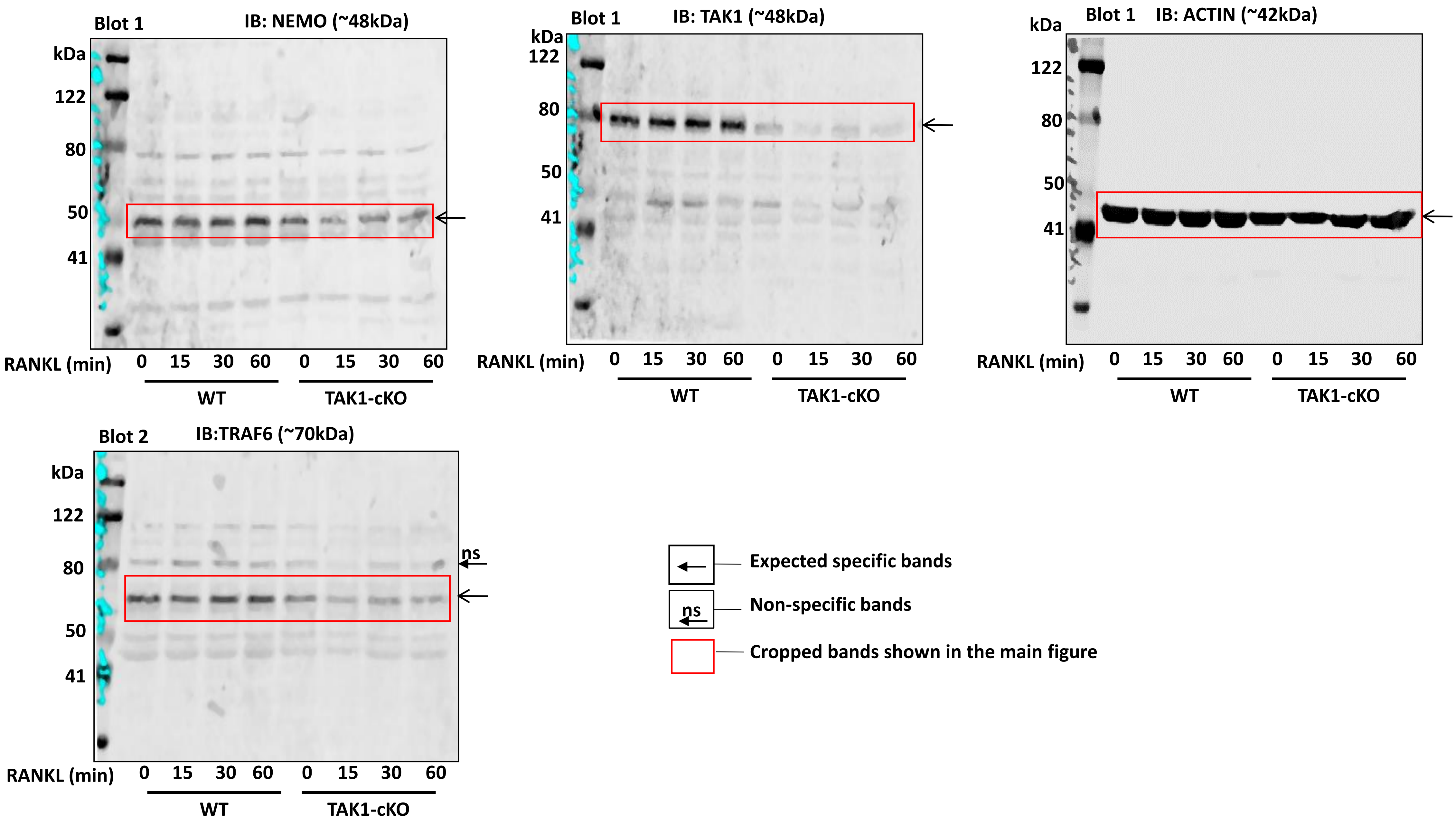
Blot 1
IB: NEMO (~48kDa)
kDa
122
80
50
41
RANKL (min)
0 15 30 60 0 15 30 60
WT
TAK1-cKO
Blot 1
IB: TAK1 (~48kDa)
kDa
122
80
50
41
RANKL (min)
0 15 30 60 0 15 30 60
WT
TAK1-cKO
Blot 1
IB: ACTIN (~42kDa)
kDa
122
80
50
41
RANKL (min)
0 15 30 60 0 15 30 60
WT
TAK1-cKO
Blot 2
IB:TRAF6 (~70kDa)
kDa
122
80
50
41
ns
RANKL (min)
0 15 30 60 0 15 30 60
WT
TAK1-cKO
Expected specific bands
ns
Non-specific bands
Cropped bands shown in the main figure

Figure S.1C

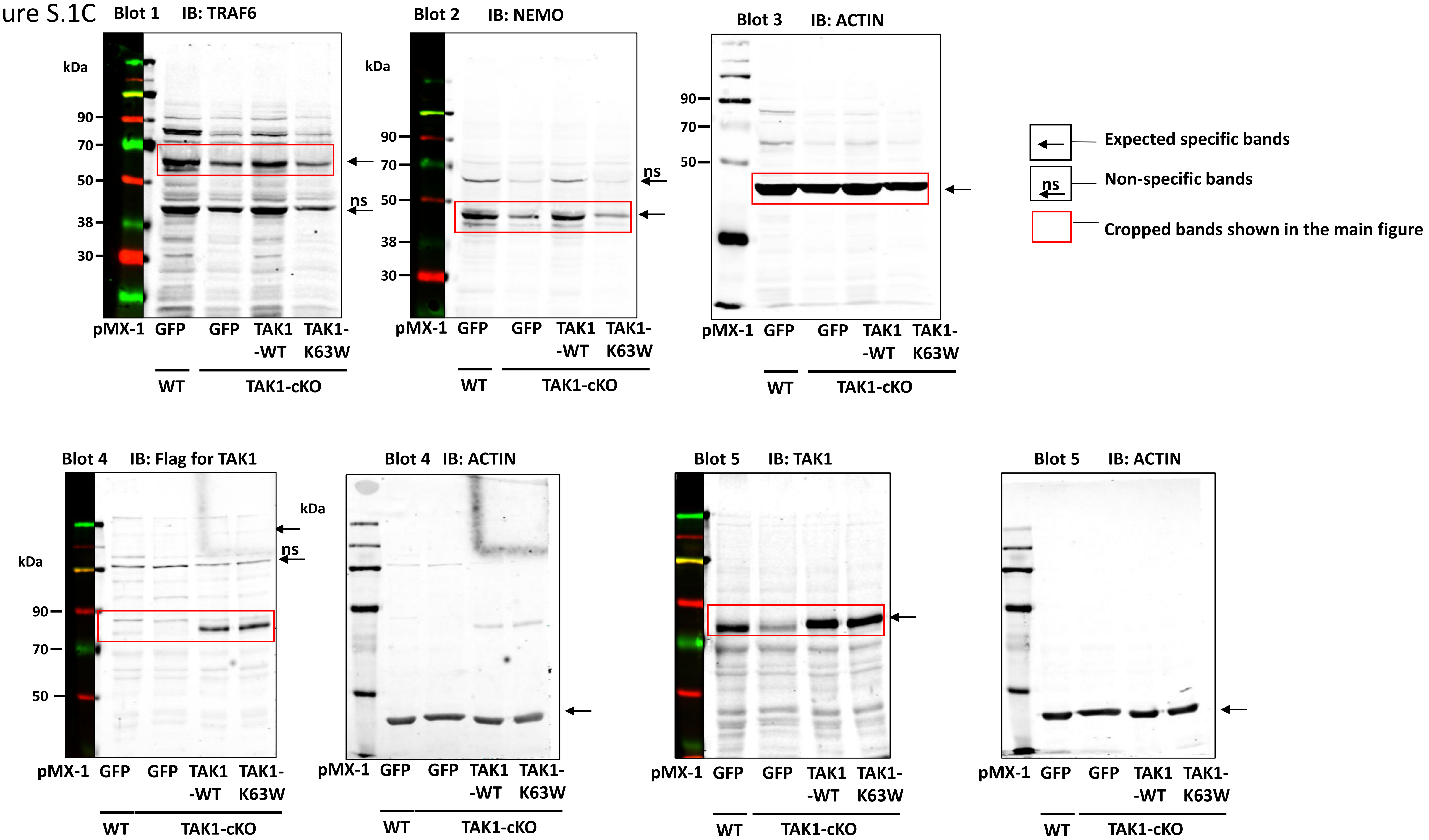
Blot 1 IB: TRAF6
kDa
90
70
50
38
30
ns
pMX-1 GFP GFP TAK1-WT TAK1-K63W
WT TAK1-cKO
Blot 2 IB: NEMO
kDa
90
70
50
38
30
ns
pMX-1 GFP GFP TAK1-WT TAK1-K63W
WT TAK1-cKO
Blot 3 IB: ACTIN
90
70
50
pMX-1 GFP GFP TAK1-WT TAK1-K63W
WT TAK1-cKO
Expected specific bands
ns
Non-specific bands
Cropped bands shown in the main figure
Blot 4 IB: Flag for TAK1
kDa
ns
kDa
90
70
50
pMX-1 GFP GFP TAK1-WT TAK1-K63W
WT TAK1-cKO
Blot 4 IB: ACTIN
pMX-1 GFP GFP TAK1-WT TAK1-K63W
WT TAK1-cKO
Blot 5 IB: TAK1
pMX-1 GFP GFP TAK1-WT TAK1-K63W
WT TAK1-cKO
Blot 5 IB: ACTIN
pMX-1 GFP GFP TAK1-WT TAK1-K63W
WT TAK1-cKO

Figure S.2K

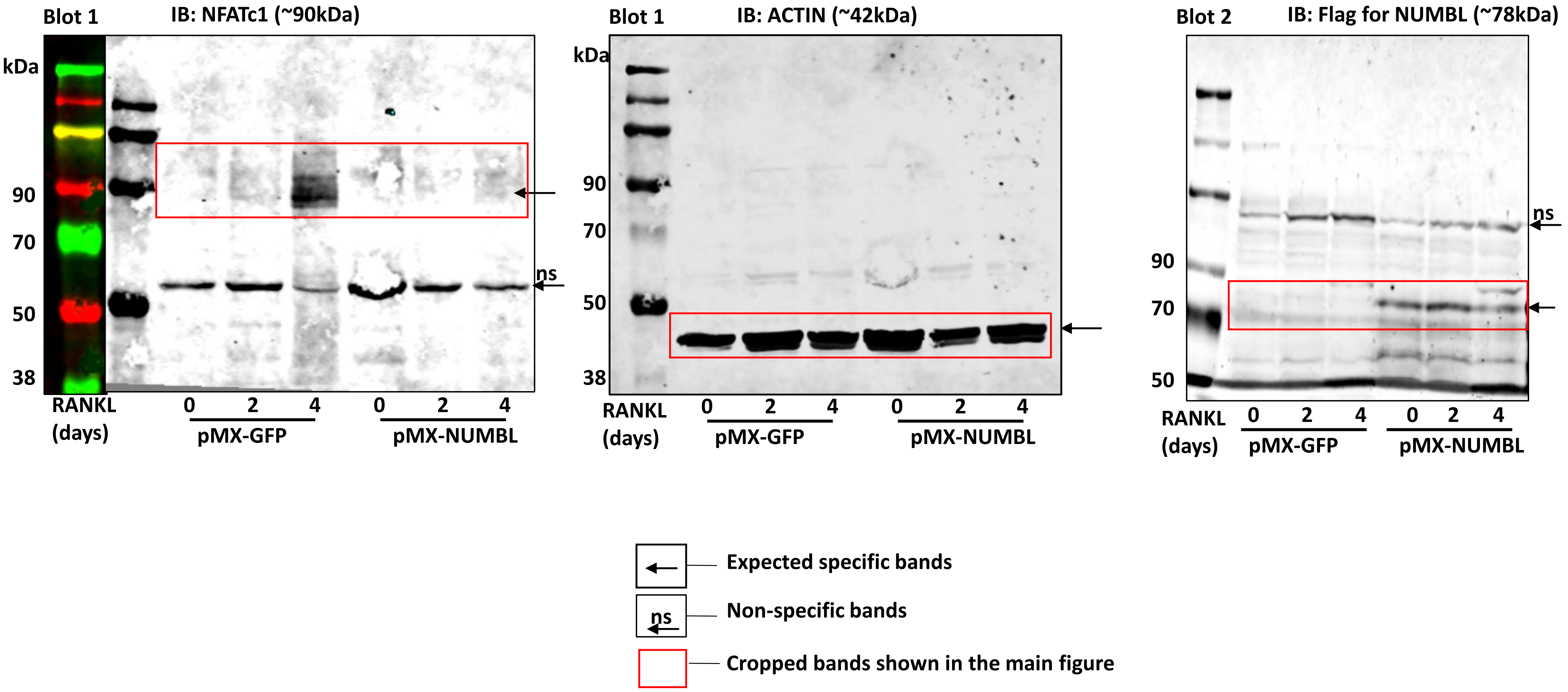
Blot 1
IB: NFATc1 (~90kDa)
kDa
90
70
50
38
ns
RANKL
(days)
0
2
4
0
2
4
pMX-GFP
pMX-NUMBL
Blot 1
IB: ACTIN (~42kDa)
kDa
90
70
50
38
RANKL
(days)
0
2
4
0
2
4
pMX-GFP
pMX-NUMBL
Blot 2
IB: Flag for NUMBL (~78kDa)
ns
90
70
50
RANKL
(days)
0
2
4
0
2
4
pMX-GFP
pMX-NUMBL
Expected specific bands
ns
Non-specific bands
Cropped bands shown in the main figure

Figure S.5A

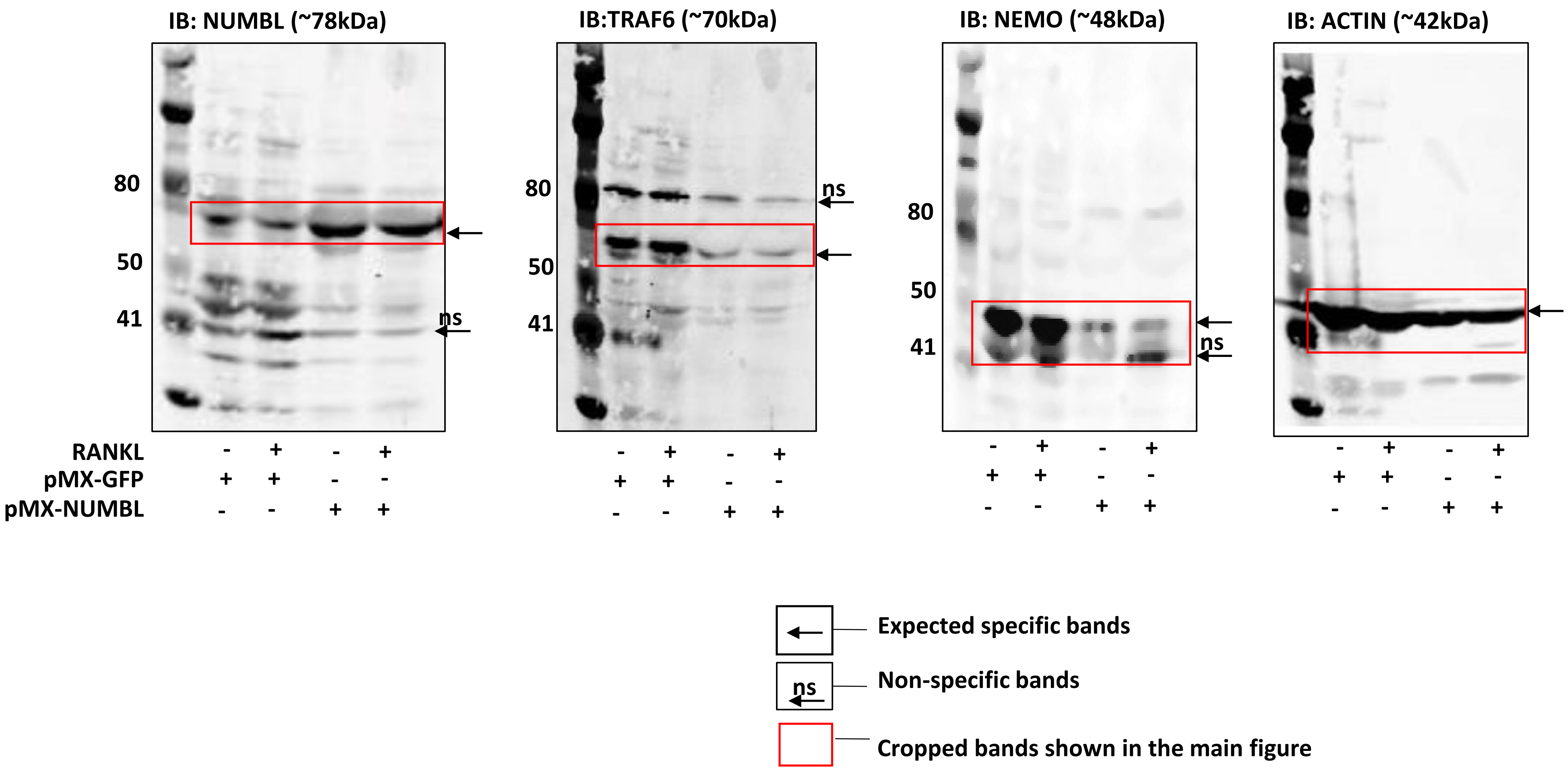

IB: NUMBL (~78kDa)
IB:TRAF6 (~70kDa)
IB: NEMO (~48kDa)
IB: ACTIN (~42kDa)
80
50
41
ns
RANKL - + - +
pMX-GFP + + - -
pMX-NUMBL - - + +
Expected specific bands
Non-specific bands
Cropped bands shown in the main figure

Figure S.5B

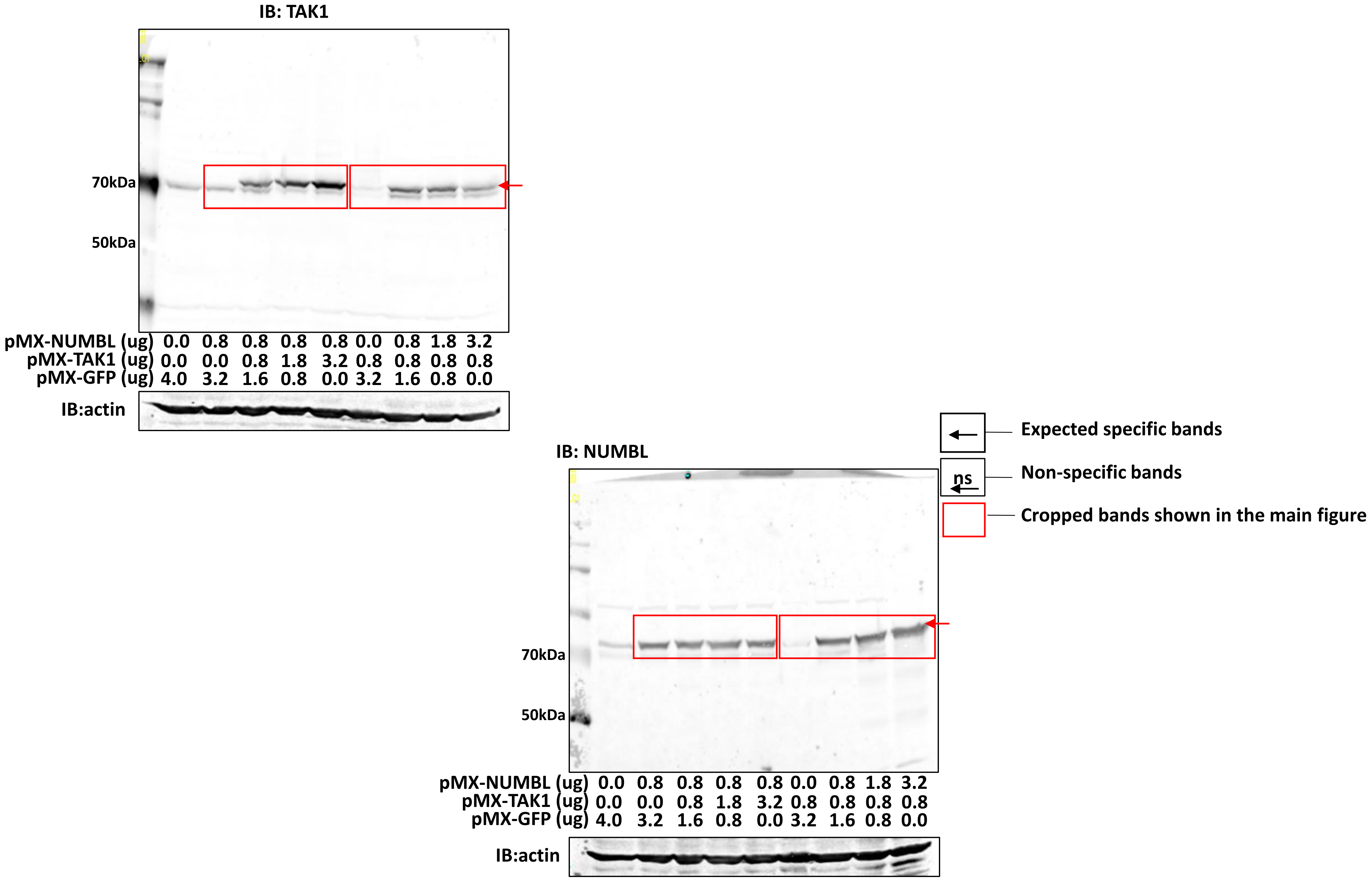
IB: TAK1
70kDa
50kDa
pMX-NUMBL (ug) 0.0 0.8 0.8 0.8 0.8 0.0 0.8 1.8 3.2
pMX-TAK1 (ug) 0.0 0.0 0.8 1.8 3.2 0.8 0.8 0.8 0.8
pMX-GFP (ug) 4.0 3.2 1.6 0.8 0.0 3.2 1.6 0.8 0.0
IB:actin
IB: NUMBL
70kDa
50kDa
pMX-NUMBL (ug) 0.0 0.8 0.8 0.8 0.8 0.0 0.8 1.8 3.2
pMX-TAK1 (ug) 0.0 0.0 0.8 1.8 3.2 0.8 0.8 0.8 0.8
pMX-GFP (ug) 4.0 3.2 1.6 0.8 0.0 3.2 1.6 0.8 0.0
IB:actin
Expected specific bands
ns
Non-specific bands
Cropped bands shown in the main figure

Figure S.5C

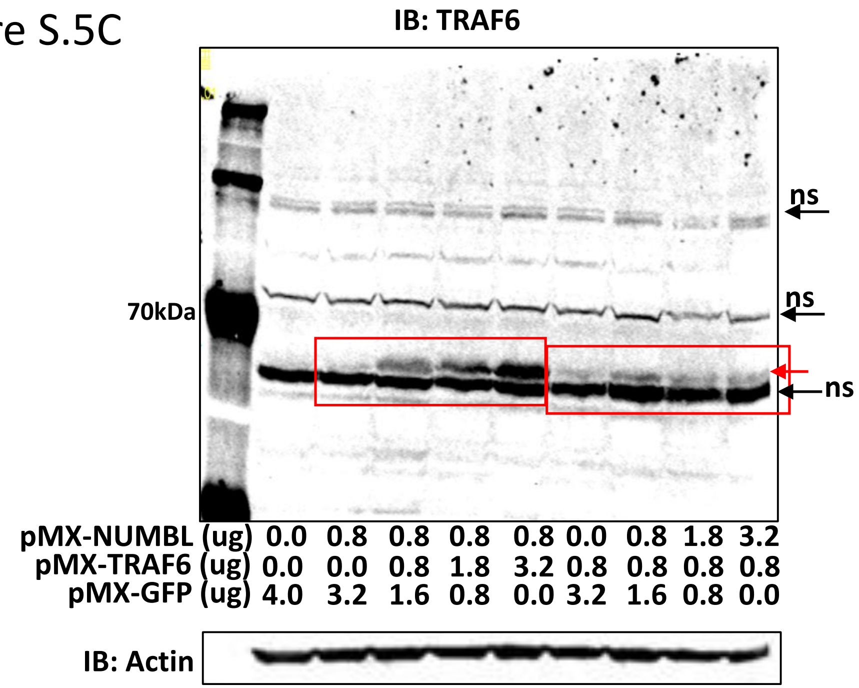
IB: TRAF6
ns
ns
70kDa
ns
pMX-NUMBL (ug) 0.0 0.8 0.8 0.8 0.8 0.0 0.8 1.8 3.2
pMX-TRAF6 (ug) 0.0 0.0 0.8 1.8 3.2 0.8 0.8 0.8 0.8
pMX-GFP (ug) 4.0 3.2 1.6 0.8 0.0 3.2 1.6 0.8 0.0
IB: Actin

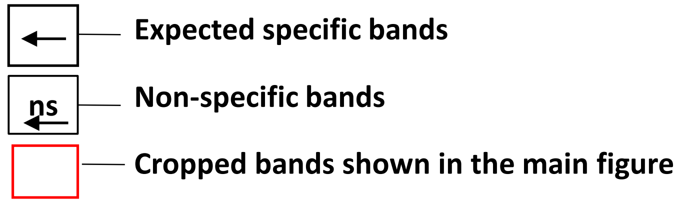
Expected specific bands
ns
Non-specific bands
Cropped bands shown in the main figure

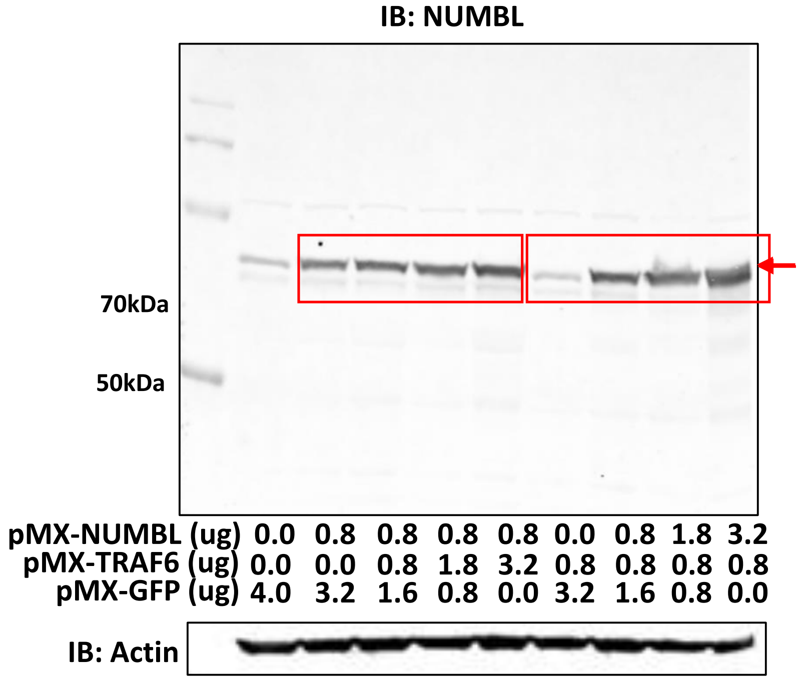
IB: NUMBL
70kDa
50kDa
pMX-NUMBL (ug) 0.0 0.8 0.8 0.8 0.8 0.0 0.8 1.8 3.2
pMX-TRAF6 (ug) 0.0 0.0 0.8 1.8 3.2 0.8 0.8 0.8 0.8
pMX-GFP (ug) 4.0 3.2 1.6 0.8 0.0 3.2 1.6 0.8 0.0
IB: Actin

Figure S.5D

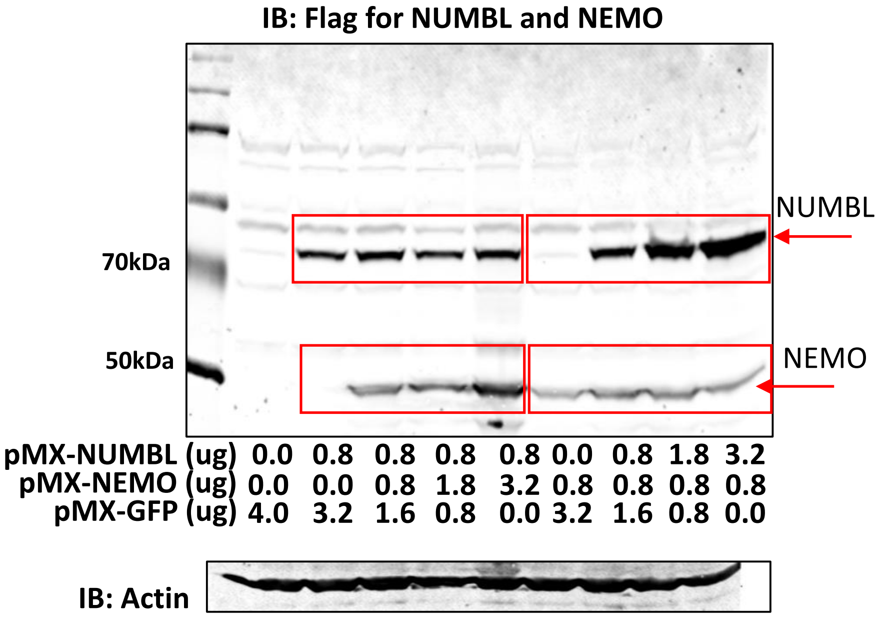

Expected specific bands

ns Non-specific bands

Cropped bands shown in the main figure

Figure S.5E

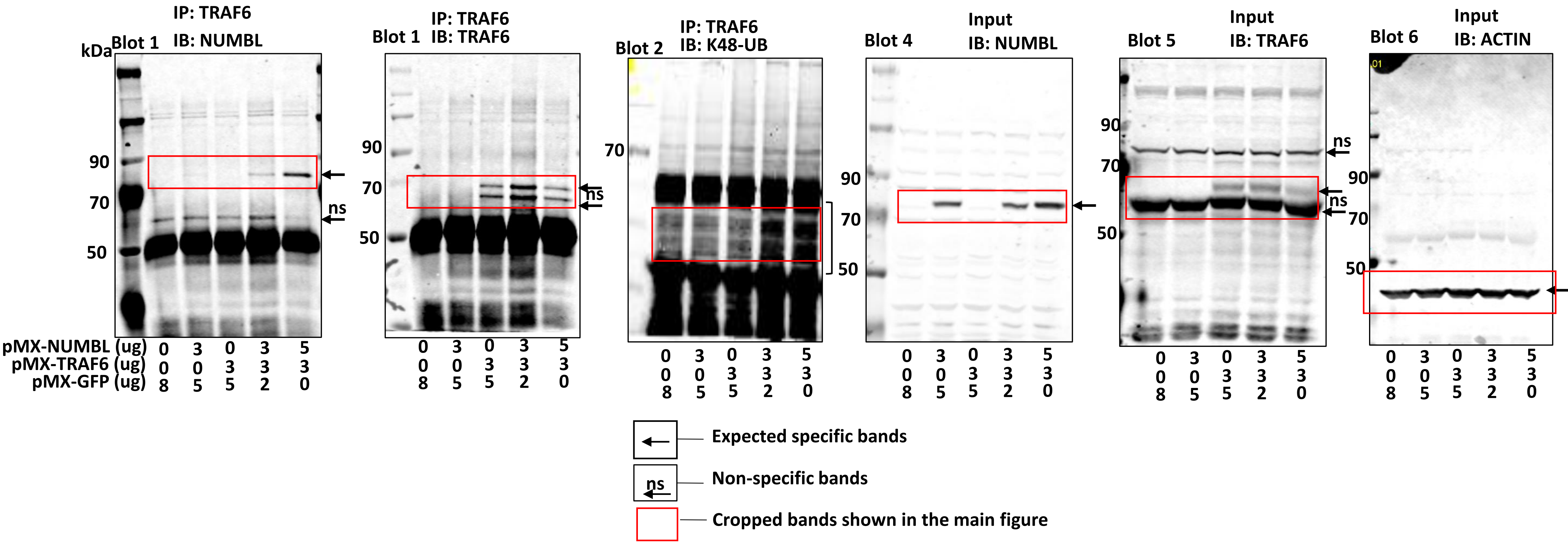

IP: TRAF6
IB: NUMBL
kDa
Blot 1
90
70
50
ns
Blot 1
IP: TRAF6
IB: TRAF6
90
70
50
ns
Blot 2
IP: TRAF6
IB: K48-UB
70
90
70
50
Blot 4
Input
IB: NUMBL
Blot 5
Input
IB: TRAF6
90
70
50
ns
ns
Blot 6
Input
IB: ACTIN
90
70
50
pMX-NUMBL (ug) 0 3 0 3 5
pMX-TRAF6 (ug) 0 0 3 3 3
pMX-GFP (ug) 8 5 5 2 0
Expected specific bands
ns
Non-specific bands
Cropped bands shown in the main figure

Figure S.5F

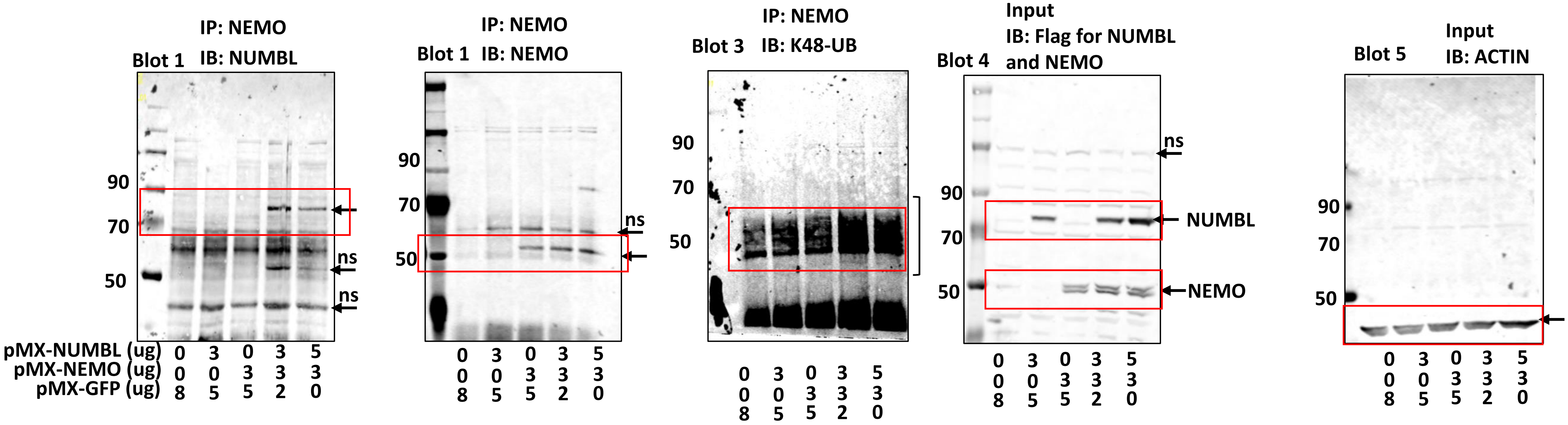

Note: For Blot 4 the membrane was blotted using anti-flag which recognizes both NUMBL and NEMO.

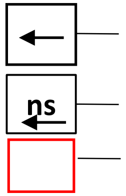

Expected specific bands

Non-specific bands

Cropped bands shown in the main figure
